# Integrin-linked kinase (ILK) expression in human colon cancer

**DOI:** 10.1038/sj.bjc.6601482

**Published:** 2003-12-09

**Authors:** V Bravou, G Klironomos, E Papadaki, D Stefanou, J Varakis

**Affiliations:** 1 Department of Anatomy, School of Medicine, University of Patras, Patras 26500, Greece; 2 Department of Pathology, General Hospital ‘Agios Andreas’, Patras 26335, Greece; 3 Department of Pathology, School of Medicine, University of Ioannina, Ioannina 45110, Greece

**Sir**,

In a recent study, Marotta *et al* (2003) reported integrin-linked kinase (ILK) overexpression and dysregulation of ILK signalling in sporadic human colon cancer. It was concluded that the dysregulation of ILK signalling is an important early event in the development of the disease.

We studied ILK expression in 84 human colorectal tumours (four adenomas and 80 carcinomas) by immunohistochemistry in order to assess whether ILK is involved in the development and progression of human colorectal carcinoma. Paraffin-embedded tissue samples were retrieved from the files of the Departments of Pathology, ‘Agios Andreas’ General Hospital, Patras, Greece and University Hospital of Ioannina, Ioannina, Greece. Clinicopathologic parameters were obtained from the pathology reports. Carcinomas were graded as: well, moderately and poorly differentiated on the basis of the degree of gland formation and staged according to the Astler Coller staging system. Immunohistochemical analysis was carried out using a standard streptavidin–biotin–peroxidase technique. Primary polyclonal anti-ILK antibody was obtained from Upstate Biotechnology (dilution 1 : 500), and immunodetection was performed with StrAvigen Multilink Immunodetection system B-SA (Biogenex) using DAB as the chromogen. Negative and positive controls were used in the study. The immunostaining intensity was evaluated by light microscopy and scored as negative (−), weak (+), moderate (++) and strong (+++). Statistical analysis was performed with SPSS 10 for Windows. Relationships between ILK expression and clinicopathologic parameters were evaluated by one-way ANOVA and Tukey test *post-hoc* analysis. *P*-values<0.05 were considered to be significant.

**Figure 1:**
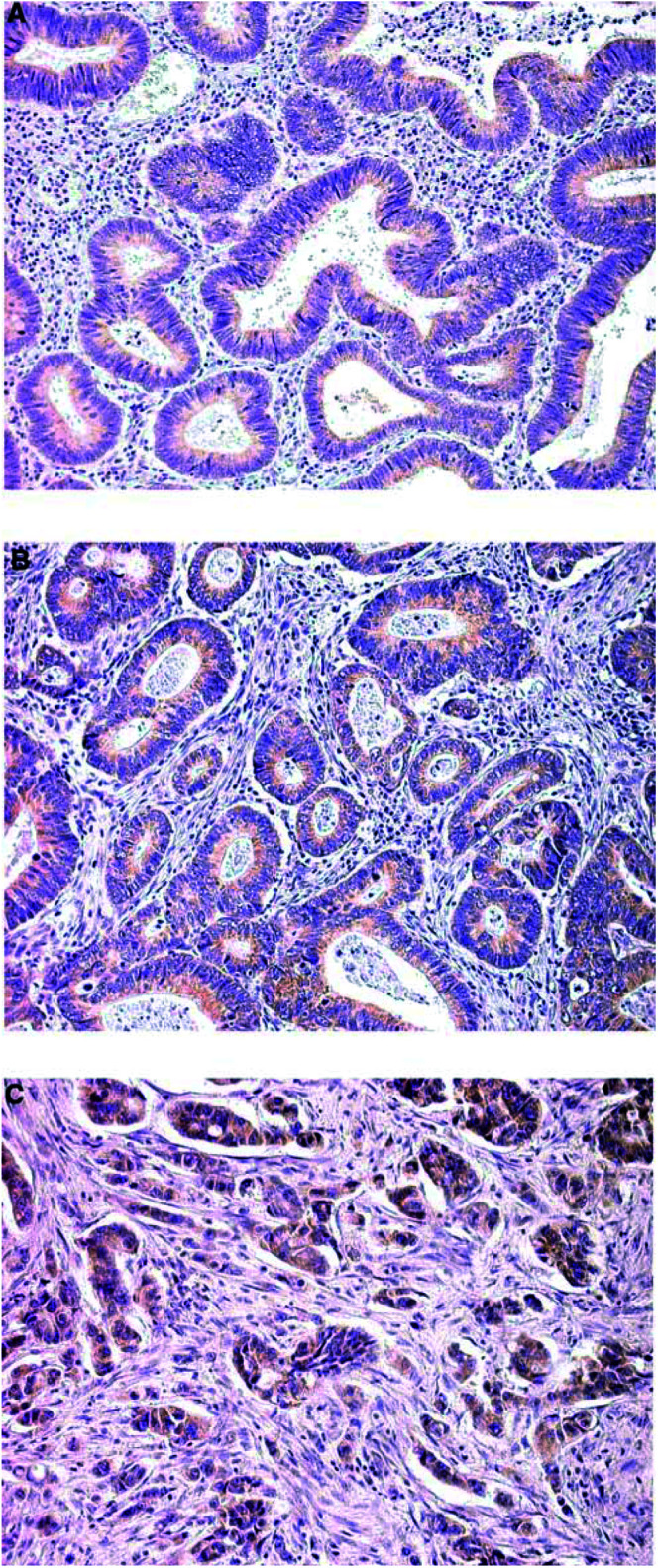
Integrin-linked kinase expression in human colorectal tumours. (**A**) Normal colonic epithelium with no ILK immunoreactivity (× 200). (**B**) Integrin-linked kinase-positive cancerous crypts compared with ILK-negative normal crypts (× 200). (**C**, **D**) Weak (+) immunostaining in an adenoma and an *in situ* carcinoma, respectively (× 200). (**E**, **F**) Invasive carcinomas demonstrating strong (+++) immunostaining (× 200).

**Figure 2:**
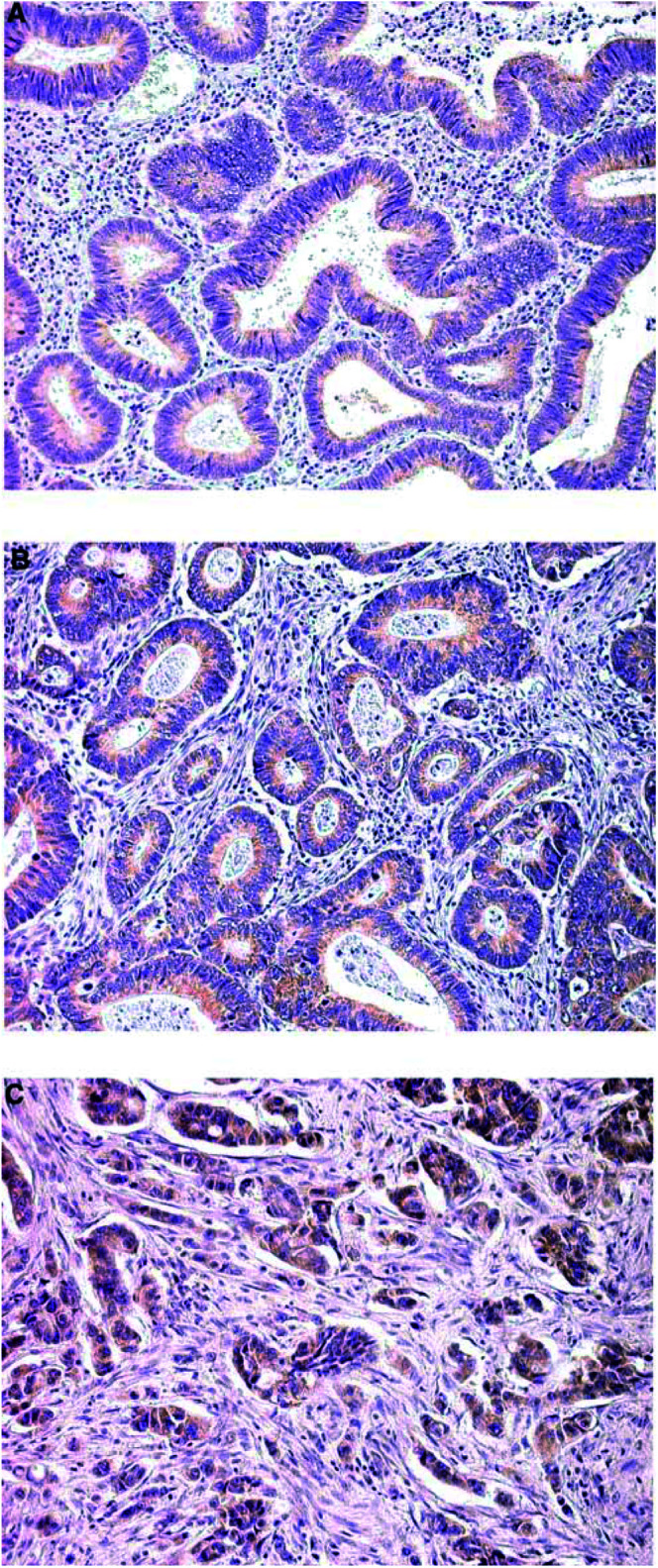
Integrin-linked kinase expression increases with tumour grade. (**A**) A well-differentiated colorectal carcinoma demonstrating weak (+) ILK expression (× 200). (**B**) Moderate levels of expression (++) in a moderately differentiated tumour (× 200). (**C**) High levels of expression (+++) in a poorly differentiated neoplasm (× 200).

There was no ILK immunoreactivity in the normal colonic epithelium (Figure 1A, B

), while the majority of adenomas (75%) and *in situ* carcinomas (83.3%) were ILK positive (Figure 1C, D). All invasive carcinomas were positive (Figure 1E, F). The levels of expression were significantly higher in invasive compared with noninvasive lesions (*P*<0.001). The intensity of ILK expression was also correlated with the depth of invasion (*P*<0.001), presence of lymph node metastasis (*P*<0.01), tumour grade (*P*<0.001) (Figure 2

) and overall staging (*P*<0.001) (Table 1

**Table 1:** Integrin-linked kinase expression in human colorectal tumours. Correlation with clinicopathologic parameters

		**ILK expression**								
		**−**		**+**		**++**		**+++**		
	***N***	***n***	**%**	***n***	**%**	***n***	**%**	***n***	**%**	***P*-value^a^**
Adenomas total	4	1	(25)	3	(75)	0	(0)	0	(0)	
Carcinomas total	80	2	(2.5)	23	(28.7)	40	(50)	15	(18.8)	
Invasive	68	0	(0)	18	(26.5)	35	(51.5)	15	(22)	<0.001
*In situ*	12	2	(16.6)	5	(41.7)	5	(41.7)	0	(0)	
*Depth of invasion*										<0.001
Tis	12	2	(16.6)	5	(41.7)	5	(41.7)	0	(0)	
T1+T2	8	0	(0)	5	(62.5)	3	(37.5)	0	(0)	
T3+T4	60	0	(0)	13	(21.7)	32	(53.3)	15	(25)	
Lymph node metastasis	35	0	(0)	7	(20)	16	(45.7)	12	(34.3)	<0.01
										
*Grade*										<0.001
Well differentiated	16	2	(12.5)	8	(50)	6	(37.5)	0	(0)	
Moderately differentiated	37	0	(0)	10	(27)	21	(56.8)	6	(10.2)	
Poorly differentiated	27	0	(0)	5	(18.5)	13	(48.2)	9	(33.3)	
										
*Astler Coller stage*										<0.001
A	12	2	(16.6)	5	(41.7)	5	(41.7)	0	(0)	
B1	8	0	(0)	5	(62.5)	3	(37.5)	0	(0)	
B2	25	0	(0)	6	(24)	16	(64)	3	(12)	
C1	0	0	(0)	0	(0)	0	(0)	0	(0)	
C2	35	0	(0)	7	(20)	16	(45.7)	12	(34.3)	

a One-way ANOVA. *P*-value<0.05 statistically significant.

). In all positive lesions, >90% of tumour cells were stained (diffuse pattern) and ILK immunostaining was confined to the cytoplasm.

Our results seem to indicate that, in addition to being involved in the initiation of colon carcinogenesis, as suggested by Marotta *et al* (2003), ILK may also be implicated in the progression, invasiveness and metastatic potential of colorectal cancer. Thus, ILK may prove to be a useful prognostic marker for these tumours.
